# Very long-chain acyl-CoA dehydrogenase deficiency revisited: a retrospective genotype–phenotype analysis in a Saudi tertiary center

**DOI:** 10.3389/fgene.2025.1584817

**Published:** 2025-05-30

**Authors:** Suzan Suliman Alhumaidi, Fahad Abdulrahman Algaeed, Meshari Fayez Aladhadh, Sara Abdulrahman Alkaff

**Affiliations:** Genetics and Metabolic Department, King Saud Medical City, Riyadh, Saudi Arabia

**Keywords:** clinical characteristics, clinical, molecular, Saudi Arabia, VLCAD deficiency, genotype-phenotype correlation, newborn screening

## Abstract

**Introduction:**

In this retrospective study, we analyzed clinical, biochemical, and genetic data and examined correlations between prevalent variants and outcomes of very long-chain acyl-CoA dehydrogenase (VLCAD) deficiency.

**Methods:**

Patients with VLCAD deficiency confirmed through molecular genetic testing at King Saud Medical City, Riyadh, Saudi Arabia, between 2016 and 2023 were included. Patients presented in the neonatal period with abnormal newborn screening and with metabolic decompensation and biochemical abnormalities clinically.

**Results:**

VLCAD deficiency was confirmed in 14 children. The mean age at presentation was 5.6 days. Clinically, 10 of the 14 patients presented with rhabdomyolysis. Hepatomegaly was observed in 9, cardiomyopathy in 7, and hypoglycemia in seven patients. In total, three variants were detected in the 14 patients: c.1310A>C (p.Glu437Ala) in 2; c.134C>A (p.Ser45X) in 6; and c.65C>A (p.Ser22Ter) in six patients. Currently, 12 patients are alive, whereas two have died. No significant relationship was identified between genotype and survival (*P* = 0.719). Variant C.1310A was associated with an excellent prognosis. Unlike those in other studies, variants c.65C>A and c.134C>A were associated with poor outcomes and early presentation with metabolic decompensation.

**Discussion:**

Long-term, prospective studies integrating metabolic profiling, functional assays, and multi-omics approaches will be essential to unravel the complex interplay between genetic variants and clinical expression and prognostic outcomes in VLCAD deficiency.

## 1 Introduction

Very long-chain acyl-CoA dehydrogenase (VLCAD) deficiency is an autosomal recessive fatty acid oxidation disorder with a markedly variable time and nature of initial clinical presentation, with associated phenotypes varying depending on the clinical presentation. Based on the severity and onset of clinical symptoms and presentations, this disorder has been classified into three broad phenotypes: the severe early-onset type, characterized by severe metabolic decompensation and cardiomyopathic involvement; the milder early-childhood type, associated with features such as varying degrees of hepatomegaly, musculoskeletal rhabdomyolysis, and intermittent hypoglycemia; and the adulthood late-onset type (Leslie and Saenz-Ayala, 2022). The global incidence of VLCAD deficiency varies from 1:30,000 to 1:100,000 live births. In Saudi Arabia, an estimated incidence of 1:37,000 live births has been reported (Alfadhel et al., 2016).

The presentation of VLCAD deficiency is heterogeneous, thereby making the diagnosis challenging. Confirmatory diagnostic tests are recommended when the clinical presentation and biomolecular markers suggest possible VLCAD deficiency. Symptomatology is predominantly associated with critical energy deficits owing to a lack of fatty acid oxidation and toxic metabolite accumulation. Patients with VLCAD deficiency have various levels of disease severity at different ages of onset. Despite the described heterogeneity, the phenotype–genotype correlations have been elaborated (Hesse et al., 2018).

Newborn screening has improved the detection rate of patients with fatty acid oxidation defects, allowing for early intervention; whole-exome sequencing is likely the most readily available approach to complete the mapping of the diagnostic workup (Marsden et al., 2021). Numerous pathogenic variants of long-chain fatty acid oxidation genes have been documented, including truncating, frameshift, and missense variants. Moreover, variants in specific populations are associated with numerous specific clinical presentations and different timing of symptom onset. VLCAD deficiency in Saudi Arabia is frequently related to homozygous nonsense variants (c.65C>A: p. Ser22Ter) in exon two of acyl-CoA dehydrogenase very long-chain (*ACADVL*) and has been associated with an early-onset phenotype. Other populations have high allele frequency-specific variants that display variable phenotypes (Obaid et al., 2018).

Despite constraints, the screening of newborns with an appropriate level of suspicion has outlined favorable prognostics for those with fatty acid oxidation defects, enabling clinicians treating newborns with such defects to employ an immediate action plan, recommend necessary lifestyle alterations, adopt management strategies tailored to the specific fatty acid oxidation defects, and provide adequate counseling to the primary caregivers (Spiekerkoetter et al., 2003). Predicting disease presentations and patterns is the major challenge encountered by many clinicians, with numerous studies focusing on evaluating phenotypic correlations. Genetic analysis is acknowledged for its potential prognostic merit, and future studies may validate genotype–phenotype relationships (Evans et al., 2016). *ACADVL* has numerous variants, with several factors affecting disease presentation. However, many variants, mainly found in the Western world, are less prevalent in the Saudi Arabian population (Pena et al., 2016; Obaid et al., 2018).

Determining precise associations between genotypes and phenotypes is challenging owing to the presence of multiple factors that can affect the disease progression and interact in diverse ways (Olpin, 2013). The identification of compounds unique to early- and late-onset alleles—causing symptoms in infancy or early childhood to adulthood—and reports of different phenotypes—varying from newborn death to symptom free adolescents and adults—emphasize the importance of further studying correlations of different variants with clinical findings and age-of-onset presentations (Merritt et al., 2018).

Initial evaluation and screening tools, biochemical examination, and acylcarnitine analysis are valuable for the diagnosis of VLCAD deficiency. Acylcarnitine level is usually abnormal during a metabolic crisis and within the standard acceptable limit between episodes. Hypoketotic hypoglycemia, high anion gap metabolic acidosis, an abnormal liver profile, and elevated creatinine phosphokinase and plasma ammonia levels are common biochemical abnormalities (Rovelli et al., 2019). Comprehensive and long-term evaluations have revealed late-onset presentations of residual enzyme activity of VLCAD, resulting in symptoms among individuals across a spectrum of age groups (from children to adults); however, the amount of residual enzyme varies. The common variant p. Val283Ala expresses itself in a way that concurs with the above findings (Diekman et al., 2015). One study has revealed that severe cases are associated with the absence of residual enzyme activity, usually resulting from compound heterozygosity for null variants or homozygosity (Andresen et al., 1999). The efficacy of the newborn screening procedure in identifying patients with the mild form of the disease remains to be determined. Additionally, the question of whether symptoms or phenotypes of the mild disease state become more apparent in older children presenting as late-onset variants remains unanswered. Observing patients beyond their childhood is necessary to elucidate the morbidity associated with VLCAD deficiency (Alhashem et al., 2020).

Early assessments and evaluations and highly predictive newborn metabolic screening have beneficial effects on outcomes as they enable preemptive planning for highly probable metabolic decompensation events in patients. The predictive value of even common variants may be adversely affected by factors with unknown variables, including but not limited to environmental factors or synergistic variants influencing disease expression (Bleeker et al., 2019). In this study, we analyzed clinical, biochemical, and molecular genetic data and examined correlations between prevalent variants and outcomes of VLCAD deficiency in Saudi Arabia.

## 2 Materials and methods

### 2.1 Editorial policies and ethical considerations

The study was approved by the Institutional Research Board of King Saud Medical City [Approval No. H1RI-02-Feb23-0], ensuring ethical considerations for data confidentiality and privacy. Patient consent was not required owing to the retrospective nature of the study, which did not involve direct patient contact. Nonetheless, confidentiality was rigorously maintained, and each patient received a study number for identification.

This retrospective observational study was conducted at King Saud Medical City, Saudi Arabia. All patients included in the study underwent newborn screening, which detected abnormalities suggestive of VLCAD deficiency. Following a positive newborn screening result, molecular testing confirmed the diagnosis. Only patients with VLCAD deficiency as confirmed using genetic analysis at the Genetic/Metabolic Section were recruited for the study. Data were extracted from electronic hospital records at the Genetic/Metabolic Section, King Saud Medical City, from 2016 to 2023. The following data were collected: 1) sociodemographic data, including the date of birth, age at diagnosis, sex, and mortality status; 2) clinical symptoms and signs, including cardiomyopathy, hepatomegaly, rhabdomyolysis, and a history of metabolic decompensation; 3) biochemical data, including the levels of creatinine phosphokinase, lactic acid, hypoglycemia, and long-chain C14 acylcarnitine; 4) genetic data, including genetic variants, markers of nucleotide change, protein alteration, zygosity, variant type (frameshift, deletion, or missense), and variant classification (i.e., pathogenic, likely pathogenic, or uncertain significance).

### 2.2 Inclusion and exclusion criteria

Patients included in the study were required to have a confirmed diagnosis of VLCAD deficiency through molecular testing, identifying a mutation in *ACADVL*. Patients diagnosed with VLCAD deficiency solely through newborn screening were excluded from the study.

### 2.3 Statistical analysis

Data were analyzed using IBM SPSS software (version 2.0) provided by IBM corporation. Descriptive statistics and frequency distributions were calculated for all variables. The chi-square test was used for variable correlation, and results with *p* < 0.05 were considered statistically significant.

## 3 Results

### 3.1 Clinical features

In total, 14 patients confirmed to have VLCAD deficiency using molecular testing between 2016 and 2023 were included in this study. Currently, 12 patients are alive, whereas two have died. No significant relationship was identified between genotype and survival (*p* = 0.719). Analysis of phenotypical and clinical characteristics revealed that seven patients (50%) had cardiomyopathy, nine had hepatomegaly (64%), 10 developed rhabdomyolysis (72%), and seven had hypoglycemia (50%). Of the 14 patients, 11 were male individuals (78%) and three were female individuals (22%), and the mean age at presentation was 5.35 days (Table 1). All patients exhibited abnormal newborn screening results 14 patients (100%).

**TABLE 1:** Demographics and clinical findings.

Clinical feature	Number of patients	Percentage (%)
Sex		
• Female	11	78
• Male	3	22
Status of mortality		
• Deceased	2	14
• Alive	12	86
Cardiomyopathy	7	50
Hepatomegaly	9	64
Rhabdomyolysis	10	72
Hypoglycemia	7	50

### 3.2 Biochemical characteristics

The analysis of biochemical and molecular characteristics revealed that eight out of the 14 patients (58%) had lactic acidemia, with ammonia level ranging between 45 and 137.4 μmol/L and creatinine phosphokinase level ranging between 199 and 24,136 U/L. Results of creatine kinase analysis were not available for three patients. Furthermore, 14 patients (100%) had high long-chain C14 acylcarnitine levels.

### 3.3 Molecular characteristics

A nonsense variant was identified in 86% and a missense variant was identified in 14% of the patients. In total, three variants were detected: c.134C>A (p.Ser45x) in 6 (42%), c.65C>A (p.Ser22Ter) in 6 (42%), and c.1310A>C (p.Glu437Ala) in two patients (14%). Furthermore, in our cohort, 93% of patients were homozygous and 7% were heterozygous for *ACADVL* variants (Table 2). The parents of all 14 patients had consanguineous marriages.

**TABLE 2:** Molecular analysis.

Number of cases	Nucleotide change	Amino acid variant	Type of variant	Zygosity	References
6	NM_000018.4:c.65C>A	p.Ser22Ter	Nonsense	Homozygous	Watanabe et al. (2000)
6	NM_001270447.2:c.134C>A	p.Ser45X	Nonsense	Homozygous	Watanabe et al. (2000)
2	NM_001270447.2: c.1310A>C	p.Glu437Ala	Missense	Homozygous	Hisahara et al. (2015)

## 4 Discussion

In this study, we analyzed the clinical findings, outliers, biochemical results, and genetic and molecular profiles of 14 patients with VLCAD deficiency. Newborn screening has improved the detection of fatty acid oxidation defects, allowing for early intervention; however, given the genotype and phenotype variability, independent detection of the disease cannot predict the magnitude of clinical severity and manifestations or whether the patient will remain asymptomatic for life (Watanabe et al., 2000; Marsden et al., 2021).

In this study, all 14 patients were diagnosed with abnormal newborn screening results. Of the 14 patients, 9 (64%) had significant clinical manifestations and symptomatology during the neonatal period, which is consistent with data from studies in Western populations (Andresen et al., 1999; Pena et al., 2016).

Early detection and intervention can contribute to a favorable disease prognosis. A study conducted in Spain showed that VLCAD deficiency in 92% of patients was detected early via newborn screening, and those who received early dietary intervention remained asymptomatic, with normal biochemical marker levels, over a mean period of 30 months. Genetic analysis helps diagnose fatty acid oxidation defects owing to its efficiency in terms of accuracy and processing time. Assessment and evaluation through next-generation sequencing have made genetic analysis much more accessible in clinical practice. Although setting up a genetic analysis may be a simple task, evaluating the results is complex, particularly when dealing with novel mutations, such as missense mutations that tend to be associated with milder phenotypes, or splice and nonsense mutations that are almost truncating, frequently resulting in severe phenotypes. Nevertheless, long-chain fatty acid oxidation defects are heterogeneous diseases, and the phenotype–genotype correlations remain to be clearly defined. Severe phenotypes may be present in cases involving missense mutations and *vice versa*, reiterating the complexity and variable factors underlying such correlations (Hisahara et al., 2015; Arunath et al., 2021).

The genetic variant c.848T>C (p.Val283Ala) is a reliable predictor of late-onset muscular afflictions. However, this variant has been mainly found in Western populations (Andresen et al., 1999; Leslie and Saenz-Ayala, 2022), and multiple cohort and chart reviews did not reveal this variant in *ACDVL* in Saudi Arabian populations (Obaid et al., 2018; Alhashem et al., 2020). In our study, genetic testing was performed to confirm the diagnosis. Next-generation sequencing, copy number variant analysis, fatty acid oxidation panels, and whole-exome sequencing were used as they are the most readily available tools in our local facility to complete the diagnostic workup. A study conducted in Saudi Arabia identified a homozygous c.65C>A (p.Ser22Ter) founder nonsense mutation in exon two of *ACADVL* in 31 (83.7%) patients with VLCAD deficiency and associated it with an early-onset phenotype (Obaid et al., 2018).

In our study, three variants were detected: c.1310A>C (p.Glu437Ala) in 2 (14%), c.134C>A (p.Ser45X) in 6 (43%), and c.65C>A (p.Ser22Ter) in six patients (43%). Twelve (86%) patients survived, while 2 (14%) have died, and no significant relationship was found between the genotype and survival (*p* = 0.719). Moreover, none of the patients with the c.1310A>C (p.Glu437Ala) variant displayed any symptoms. A relevant predisposing factor that must be considered is the relatively high rate of consanguinity in the Saudi Arabian population (Merinero et al., 2018). A spectrum of variants, mostly missense, but also nonsense stop codon and frameshift variants, has been detected (Obaid et al., 2018; Alhashem et al., 2020).

In a local study involving 37 patients, the early-onset type of VLCAD deficiency in several patients was categorized under severe complications attributed to a poor prognosis and high mortality rate with the mortality rate being as high as 62%) (Alhashem et al., 2020). In another study in Saudi Arabia, the mortality rate among 14 patients was 28% (Obaid et al., 2018).

During the pre-newborn-screening era, the mortality rate was as high as 75% (Andresen et al., 1999). Among our patients, 12 (86%) are alive, whereas 2 (14%) have died. This finding could be explained by the early detection and intervention in our patients, as all of subjects were diagnosed during the neonatal period, with a mean age at diagnosis of 5.35 days. Furthermore, we observed no significant relationship between the genotype and survival. The major challenges that are expected to be encountered in early detection via early screening for fatty acid oxidation disorders are false negatives, overt magnification of benign phenotypes owing to the rarity of the disease, false positives being poorly interpreted because of lack of expertise, poor prognosis of the neonatal-onset form, and a lack of a clear prognosis with sporadic novel mutations (Arunath et al., 2021).

These challenges emphasize the importance of genetic studies and analyzing correlations of variant associations with clinical findings and age-of-onset presentations (Olpin, 2013; Merritt et al., 2018). Even common variants may be expressed in an abstruse manner in response to unknown factors, such as, but not limited to, environmental factors and synergistic variants that influence disease manifestation. As molecular diagnostics become more widely available and are brought into effective action, longitudinal scientific evaluations of clinical findings and manifestations and frequent advancements in biochemical and molecular testing will prove valuable in defining the role of the genotype in patients with long-chain fatty acid oxidation defects, and observations beyond childhood are necessary to fully understand the effect of VLCAD deficiency on morbidity (Andresen et al., 1999; Bleeker et al., 2019; Rovelli et al., 2019; Yamada and Taketani, 2019).

An interesting finding in our study was the variability between expected and observed clinical severity associated with certain ACADVL variants, particularly c.65C>A and c.134C>A. While these variants have been reported in other cohorts with variable outcomes, our cohort exhibited early and severe metabolic decompensation. This discordance may be influenced by several biological factors, including the differences in residual VLCAD enzyme activity, the presence of modifying genetic elements, or synergistic epigenetic influences that alter gene expression. Additionally, environmental stressors such as fasting, infections, or metabolic demands could exacerbate phenotypic severity in genetically susceptible individuals. Variability has been observed in studies by (Alhashem et al., 2020; Obaid et al., 2018), emphasizing the complexity of phenotype prediction in fatty acid oxidation disorders.

In conclusion, in our cohort, the first sign of significant presentation was in the neonatal period, either by newborn screening or clinically, with metabolic decompensation and biochemical abnormalities. The implementation of clinical diagnostics, metabolic screening, and molecular genetic testing led to early diagnosis, which aided in establishing a starting point in care and prognostication strategies to tackle possible complications. In our study cohort, variant c.1310A>C was associated with a favorable prognosis. In contrast, variants c.65C>A and c.134C>A were associated with poor clinical outcomes; they were identified early because of clinical manifestations and metabolic decompensation, in contrast to findings in other studies. Future studies employing functional characterization of mutations, genotype-based clinical risk stratification, transcriptomic profiling, and multi-omics integration will be crucial in clarifying these relationships and informing genotype-driven prognostication.

## 5 Limitation

This study is subject to several limitations. As a retrospective analysis, it may be affected by selection and information biases, potentially influencing the collected data. The study was conducted at a single tertiary center with a relatively small cohort (n = 14) it is important to note, however, that VLCAD deficiency remains a rare metabolic disorder in our population, and the small sample size reflects the low prevalence of this condition in clinical practice, which limits the generalizability of the findings to broader populations. Additionally, the reliance on electronic health records further introduces the possibility of missing or inconsistently documented information, particularly in earlier or less severe cases. To address this, we applied strict inclusion and exclusion criteria requiring molecular confirmation of VLCAD deficiency to minimize the risk of misclassification and reduce data incompleteness. While the statistical methods employed were appropriate for the study objectives, the relatively limited sample size and data scope must be considered when interpreting the results.

Future prospective studies incorporating functional assays and structural modeling could help clarify these relationships and improve our understanding of disease variability.

## Data Availability

The original contributions presented in the study are included in the article/Supplementary Material, further inquiries can be directed to the corresponding author.

## References

[B1] Alfadhel M. Benmeakel M. Hossain M. A. Al Mutairi F. Al Othaim A. Alfares A. A. (2016). Thirteen-year retrospective review of the spectrum of inborn errors of metabolism presenting in a tertiary center in Saudi Arabia. Orphanet J. Rare Dis. 11, 126. 10.1186/s13023-016-0510-3 27629047 PMC5024448

[B2] Alhashem A. Mohamed S. Abdelraheem M. AlGufaydi B. Al-Aqeel A. (2020). Molecular and clinical characteristics of very-long-chain acyl-CoA dehydrogenase deficiency: a single-center experience in Saudi Arabia. Saudi Med. J. 41, 590–596. 10.15537/smj.2020.6.25131 32518924 PMC7502945

[B3] Andresen B. S. Olpin S. Poorthuis B. J. Scholte H. R. Vianey-Saban C. Wanders R. (1999). Clear correlation of genotype with disease phenotype in very-long-chain acyl-CoA dehydrogenase deficiency. Am. J. Hum. Genet. 64, 479–494. 10.1086/302261 9973285 PMC1377757

[B4] Arunath V. Liyanarachchi M. S. Gajealan S. Jasinge E. Weerasekara K. Moheb L. A. (2021). A novel mutation in ACADVL causing very long-chain acyl-coenzyme-A dehydrogenase deficiency in a South Asian pediatric patient: a case report and review of the literature. J. Med. Case Rep. 15, 441. 10.1186/s13256-021-03013-y 34465376 PMC8407922

[B5] Bleeker J. C. Kok I. L. Ferdinandusse S. van der Pol W. L. Cuppen I. Bosch A. M. (2019). Impact of newborn screening for very-long-chain acyl-CoA dehydrogenase deficiency on genetic, enzymatic, and clinical outcomes. J. Inherit. Metab. Dis. 42, 414–423. 10.1002/jimd.12075 30761551

[B6] Diekman E. F. Ferdinandusse S. van der Pol L. Waterham H. R. Ruiter J. P. Ijlst L. (2015). Fatty acid oxidation flux predicts the clinical severity of VLCAD deficiency. Genet. Med. 17, 989–994. 10.1038/gim.2015.22 25834949

[B7] Evans M. Andresen B. S. Nation J. Boneh A. (2016). VLCAD deficiency: follow-up and outcome of patients diagnosed through newborn screening in Victoria. Mol. Genet. Metab. 118, 282–287. 10.1016/j.ymgme.2016.05.012 27246109

[B8] Hazmi M. A. Al-Swailem A. R. Warsy A. S. Al-Swailem A. M. Sulaimani R. Al-Meshari A. A. (1995). Consanguinity among the Saudi Arabian population. J. Med. Genet. 32, 623–626. 10.1136/jmg.32.8.623 PMC1051637 7473654

[B9] Hesse J. Braun C. Behringer S. Matysiak U. Spiekerkoetter U. Tucci S. (2018). The diagnostic challenge in very-long chain acyl-CoA dehydrogenase deficiency (VLCADD). J. Inherit. Metab. Dis. 41, 1169–1178. 10.1007/s10545-018-0245-5 30194637

[B10] Hisahara S. Matsushita T. Furuyama H. Tajima G. Shigematsu Y. Imai T. (2015). A heterozygous missense mutation in adolescent-onset very long-chain acyl-CoA dehydrogenase deficiency with exercise-induced rhabdomyolysis. Tohoku J. Exp. Med. 235, 305–310. 10.1620/tjem.235.305 25843429

[B11] Leslie N. D. Saenz-Ayala S. (2022). in Very long-chain acyl-coenzyme A dehydrogenase deficiency” in GeneReviews^®^ Editors Adam M. P. Everman D. B. Mirzaa G. M. Pagon R. A. Wallace S. E. Bean L. J. H. (Seattle: University of Washington), 1993–2023. 20301763

[B12] Marsden D. Bedrosian C. L. Vockley J. (2021). Impact of newborn screening on the reported incidence and clinical outcomes associated with medium- and long-chain fatty acid oxidation disorders. Genet. Med. 23, 816–829. 10.1038/s41436-020-01070-0 33495527 PMC8105167

[B13] Merinero B. Alcaide P. Martín-Hernández E. Morais A. García-Silva M. T. Quijada-Fraile P. (2018). Four years’ experience in the diagnosis of very long-chain acyl-CoA dehydrogenase deficiency in infants detected in three Spanish newborn screening centers. JIMD Rep. 39, 63–74. 10.1007/8904_2017_40 28755359 PMC5953901

[B14] Merritt J. L. Norris M. Kanungo S. (2018). Fatty acid oxidation disorders. Ann. Transl. Med. 6, 473. 10.21037/atm.2018.10.57 30740404 PMC6331364

[B15] Obaid A. Nashabat M. Alfadhel M. Alasmari A. Al Mutairi F. Alswaid A. (2018). Clinical, biochemical, and molecular features in 37 Saudi patients with very long chain acyl CoA dehydrogenase deficiency. JIMD Rep. 40, 47–53. 10.1007/8904_2017_58 28980192 PMC6122013

[B16] Olpin S. E. (2013). Pathophysiology of fatty acid oxidation disorders and resultant phenotypic variability. J. Inherit. Metab. Dis. 36, 645–658. 10.1007/s10545-013-9611-5 23674167 PMC7101856

[B17] Pena L. D. van Calcar S. C. Hansen J. Edick M. J. Walsh Vockley C. Leslie N. (2016). Outcomes and genotype-phenotype correlations in 52 individuals with VLCAD deficiency diagnosed by NBS and enrolled in the IBEM-IS database. Mol. Genet. Metab. 118, 272–281. 10.1016/j.ymgme.2016.05.007 27209629 PMC4970910

[B18] Rovelli V. Manzoni F. Viau K. Pasquali M. Longo N. (2019). Clinical and biochemical outcome of patients with very long-chain acyl-CoA dehydrogenase deficiency. Mol. Genet. Metab. 127, 64–73. 10.1016/j.ymgme.2019.04.001 31031081

[B19] Spiekerkoetter U. Sun B. Zytkovicz T. Wanders R. Strauss A. W. Wendel U. (2003). MS/MS-based newborn and family screening detects asymptomatic patients with very-long-chain acyl-CoA dehydrogenase deficiency. J. Pediatr. 143, 335–342. 10.1067/S0022-3476(03)00292-0 14517516

[B20] Watanabe R. Murakami Y. Marmor M. D. Inoue N. Maeda Y. Hino J. (2000). Initial enzyme for glycosylphosphatidylinositol biosynthesis requires PIG-P and is regulated by DPM2. EMBO J. 19, 4402–4411. 10.1093/emboj/19.16.4402 10944123 PMC302040

[B21] Yamada K. Taketani T. (2019). Management and diagnosis of mitochondrial fatty acid oxidation disorders: focus on very-long-chain acyl-CoA dehydrogenase deficiency. J. Hum. Genet. 64, 73–85. 10.1038/s10038-018-0527-7 30401918

